# Fabrication of Biomass-Derived Carbon Aerogels with High Adsorption of Oils and Organic Solvents: Effect of Hydrothermal and Post-Pyrolysis Processes

**DOI:** 10.3390/ma9090758

**Published:** 2016-09-06

**Authors:** Aishu Yin, Feng Xu, Xueming Zhang

**Affiliations:** Beijing Key Laboratory of Lignocellulosic Chemistry, Beijing Forestry University, Beijing 100083, China; asyin@bjfu.edu.cn

**Keywords:** biomass, carbon aerogels, hydrothermal treatment, recyclable, adsorption oil

## Abstract

Biomass is the most plentiful and well-utilized renewable carbon resource on the earth. Direct conversion of biomass to carbon aerogel provides a promising approach to develop adsorbent materials. In the present work, the effect of presence of water during hydrothermal treatment and holding temperature during post-pyrolysis process have been investigated for the preparation of carbon aerogels (CAs) using eggplant as raw material. The results showed that the addition of water during hydrothermal treatment was advantageous for the preparation of CA samples with higher surface area and stronger hydrophobicity, resulting in superior adsorption capacities of CAs for both oil and organic solvents compared with that fabricated without the presence of water. The optimized carbon aerogel possessed higher specific surface of 249 m^2^·g^−1^ and exhibited excellent hydrophobicity with a water contact angle of 133°. The adsorption capacities of carbon aerogel for oils and organic solvents could reach 35–45 times its own weight. In addition, the adsorbed oil and organic solvents could be recovered by distillation, and the regenerated carbon aerogels samples exhibited the stable performance and outstanding reusability. Therefore, the carbon aerogel has great potential in application of oil recovery and environmental protection.

## 1. Introduction

In order to reduce water pollution and protect the environment, it is urgent to develop an economic and feasible strategy to remove the pollutants such as petroleum products and toxic organic solvents from wastewater [1]. Many conventional methods including adsorption, oil combustion, and physical diffusion have been used to recover crude oil and organic solvents. However, they either show poor efficiency or may introduce other types of containments during the cleanup procedures [2,3,4].

In recent years, much attention has been paid to developing porous materials as oil adsorbents because they could achieve oil-water separation via a simple, fast, and effective adsorption process [2]. Among these, the aerogel has been demonstrated to be a very efficient oil adsorbent due to the ultra-light and highly porous structure. However, the conventional aerogel materials possessed the drawbacks of low absorption capacities and nonselective adsorption to both water and oil. In order to solve these limitations, particular attention has been directed to the development of carbon-based aerogels, such as carbon fiber aerogel, carbon nanotube (CNT) aerogel and graphene aerogel [5,6,7]. Although carbon nanotube and graphene aerogel exhibited excellent selectivity and adsorption capacities for oil and organic solvents, the harmful and expensive precursors, complicated processes, and complex equipment involved in CNT and graphene aerogel fabrications dramatically hampered their massive production for practical applications [8]. Therefore, increasing attention has been paid to biomass-based carbon aerogels due to their advantages of low cost, rich source and nontoxicity to humans [9,10]. In general, the carbon aerogel is prepared through well-established hydrothermal carbonization (HTC), which includes hydrothermal treatment and post-pyrolysis processes [10,11]. Li et al. hydrothermally transformed winter melon into carbon aerogel with low density (0.048 g·cm^−3^), excellent hydrophobicity and good selectivity for adsorption of organic solvents and oils. Moreover, the twisted carbon fibers (TCF) aerogels with high adsorption capacity, and good recyclability have been prepared by a facile method using an economic, environmentally friendly raw material (raw cotton). The TCF aerogel possessed the high adsorption capacity up to 50–190 times its own weight.

However, to the best of our knowledge, the key role of the presence of water in the hydrothermal treatment and holding temperature in the pyrolysis for the preparation of carbon aerogels has not been investigated. Therefore, in the present work, the influence of the presence of water in hydrothermal treatment and holding temperature in pyrolysis on the fabrication of biomass-derived carbon aerogels have been studied. In addition, the fabricated carbon aerogels were characterized using scanning electron microscopy (SEM), pore size distribution and thermal stability. Moreover, the adsorption capacities for oil and organic solvents of carbon aerogels were also investigated. Considering the high performance, the obtained carbon aerogels were believed to have great potential in wastewater treatment for removing pollutants.

## 2. Results and Discussion

### 2.1. Effect of Hydrothermal Treatments and Carbonization on Microstructure

**Figure 1:**
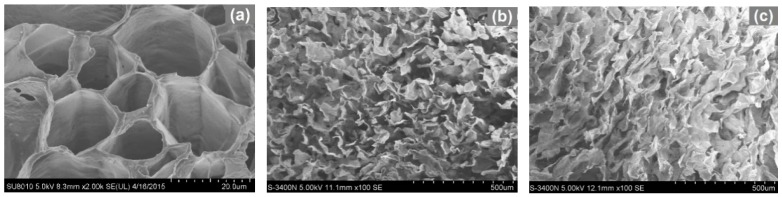

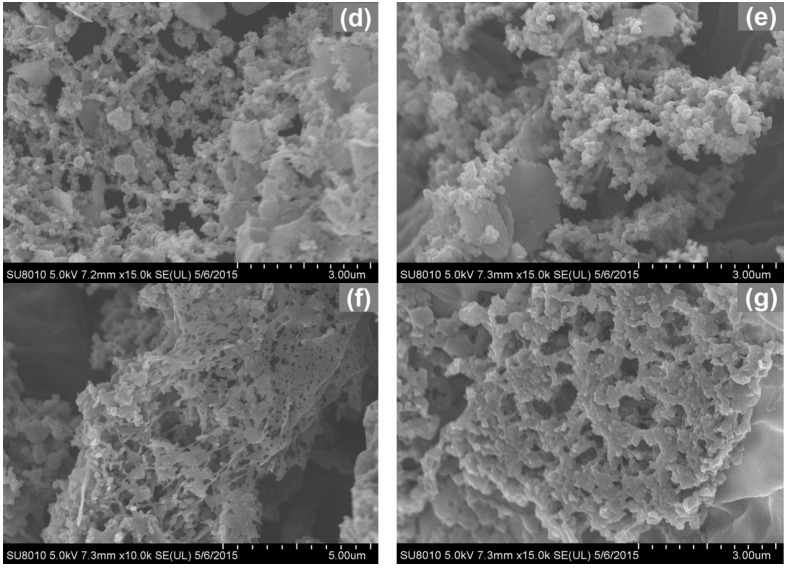
SEM images of eggplant hydrogels (EH) and carbon aerogels (CAs). (**a**) E_c_; (**b**) EH_1_; (**c**) EH_2_; (**d**) CA_1_; (**e**) CA_2_; (**f**) CA_3_; (**g**) CA_4_.

In order to investigate the microstructure changes induced by the two-step treatments, the scanning electron microscopy (SEM) was carried out to characterize the internal structure (Figure 1). In general, there are two main tissues existed in a section of eggplant. An external layer, named epicarp, appears containing the anthocyanins pigments, which provides the typical purple color of the eggplant. Under the epicarp and occupying almost the rest of the flesh fruit, a spongy and white tissue is placed, which is usually called endocarp. In the present study, only the spongy endocarp tissue was used in preparation of carbon aerogels. As shown in Figure 1a, intact tubular and reticulation structures with regular interspaces were observed, which would be helpful for the fabrication of porous aerogels. It was noted that the encocarp tissues were partially collapsed and degraded after hydrothermal treatments (Figure 1b,c). Moreover, much severe degradation was detected in sample EH_2_ (Figure 1c), which was mainly attributed to the extraction and removal of degraded products such as hemicelluloses with the addition of water during hydrothermal treatment [12]. After carbonization processing, the endocarp tissue was dramatically collapsed (Figure 1d–g), thereby, the intercellular spaces disappeared, leading to lost the spongy structure. In comparison, the samples CA_3_ and CA_4_ possessed more porous structure than that from the samples CA_1_ and CA_2_, which revealed that the presence of water during hydrothermal treatment would be advantageous for the preparation of highly porous carbon aerogels.

### 2.2. FT-IR Spectra

**Figure 2:**
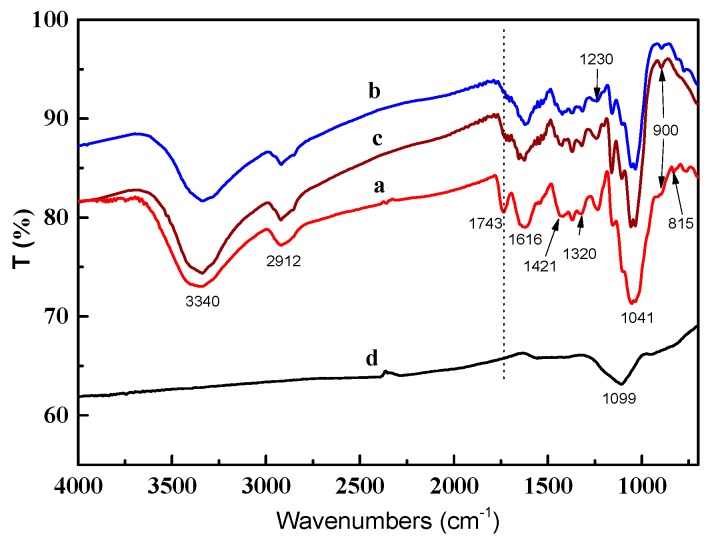
Fourier-transform infrared spectroscopy (FT-IR) of eggplant gels. (**a**) E_c_; (**b**) EH_1_; (**c**) EH_2_; (**d**) CA_1_.

In order to study and compare the structural changes taking place during hydrothermal treatment and carbonization procedures, FT-IR spectra of raw material, eggplant hydrogels and carbon aerogels were recorded as shown in Figure 2. The broad bands at 3340 cm^−1^ and 2912 cm^−1^ were originated from the stretching of –OH groups and the C–H stretching of methyl and methylene groups, respectively. The occurrence of an intensive peak at 1741 cm^−1^ was assigned to C=O stretching originated from the carbonyl and acetyl groups in hemicelluloses. Compared with raw material sample E_c_, it was noted that intensity of this peak decreased thoroughly after hydrothermal treatment (spectra b and c), which revealed that the acetyl groups in hemicelluloses fractions were removed in hydrothermal treatment. Moreover, the relative intensities of the bands for aromatic skeleton vibrations were detected at 1616 cm^−1^ and 1421 cm^−1^, indicating the presence of lignin fractions in the materials. In addition, the bands at 1320 cm^−1^ and 1230 cm^−1^ related to ring breathing with C–O stretching, and the 1320 cm^−1^ band was associated with syringyl units. The spectral pattern in the 1200–800 cm^−1^ region gives information about the polysaccharide types present. The bands at 1099 cm^−1^ could be ascribed to the C–O–C stretching vibration of vinyl C–H. Moreover, the intensive absorbance at 1041 cm^−1^ was assigned to the C–O–C stretching of glycosidic linkages, which was typical of xylans [13]. In the anomeric region (950–700 cm^−1^), a small band at 900 cm^−1^, was indicative of *β*-glycosidic linkages between the sugar units in hemicelluloses. The wavenumber at 815 cm^−1^ was attributed to sugar monomer units of mannose in glucomannans [14].

### 2.3. Thermal Analysis

**Figure 3:**
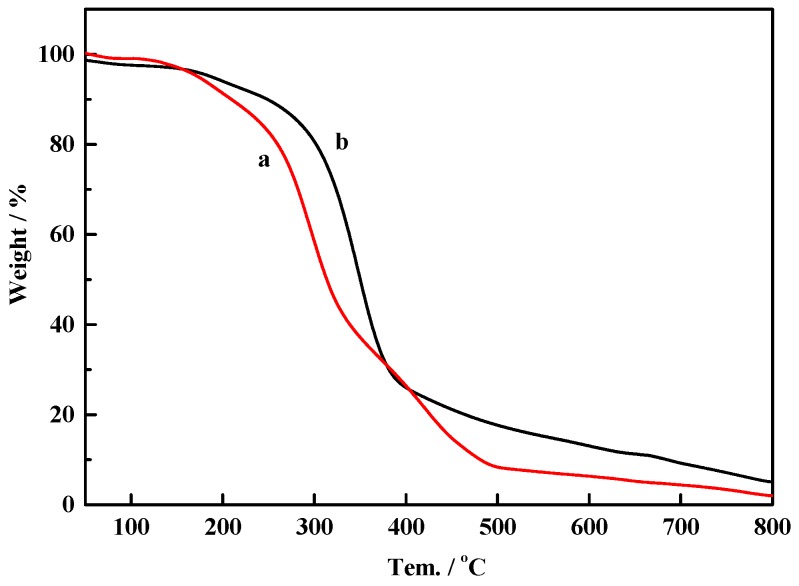
Thermogravimetric analysis (TGA) of different eggplant hydrogels. (**a**) EH_1_; (**b**) EH_2_.

The thermal behaviors of the eggplant hydrogels were comparatively characterized using thermogravimetric analysis, and the TGA curves of the samples EH_1_ and EH_2_ are shown in Figure 3. As seen from Figure 3, both TGA curves of the pyrolysis process could be divided into two weight loss stages, corresponding to the slow pyrolysis (160–280 °C) and fast pyrolysis (280–400 °C) stages, respectively. At the first stage, both of the samples EH_1_ and EH_2_ showed onset of thermal degradation at 156 °C corresponding to around 20% and 13% of the total weight losses, respectively. The weight losses could be interpreted as being due to the pyrolysis of some lower molecular weight components, such as hemicelluloses [15]. Moreover, it could be concluded that the sample EH_2_ exhibited higher thermal stability compared with sample EH_1_, which was mainly caused by the high-efficient removal of hemicelluloses during hydrothermal treatment for sample EH_2_. The maximum rate of weight loss was observed at the second stage, in which over 71% weight was pyrolyzed between 280 and 500 °C for sample EH_1_, while small amounts of EH_2_ were pyrolyzed between 280 and 400 °C.

### 2.4. Surface Area, Pore Diameter Distribution and Hydrophobicity

**Figure 4:**
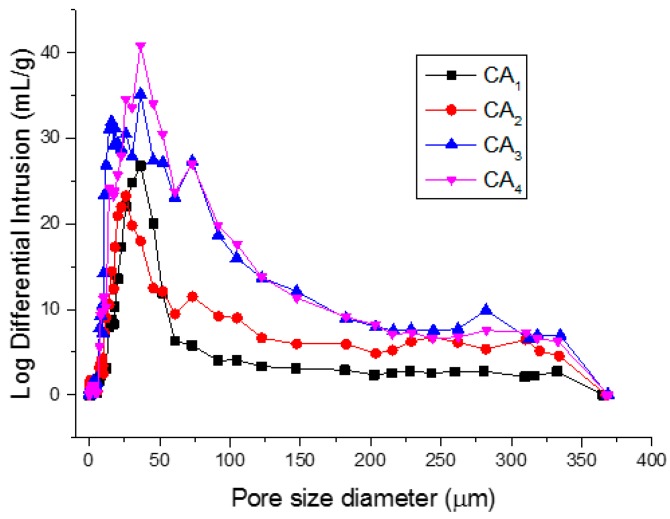
Pore size distribution of carbon aerogels.

**Figure 5:**
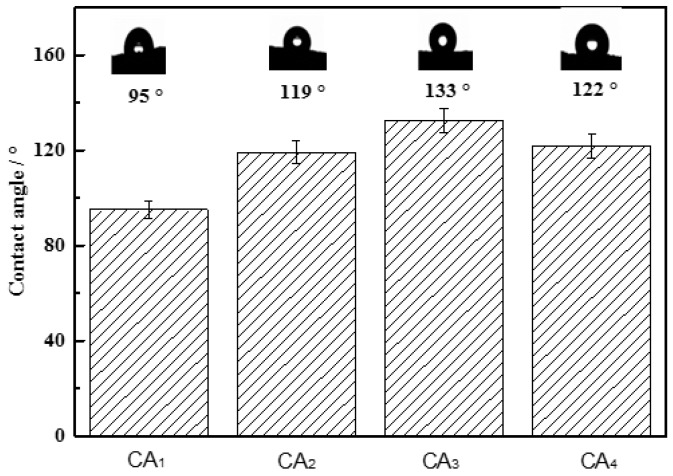
Water contact angle of carbon aerogels. The error bar in each column indicates the standard deviation.

Porosity is one of the key parameters associated with adsorption capacities. Therefore, the pore structures of carbon aerogels were characterized using nitrogen adsorption and mercury intrusion porosimetry. The Brunauer-Emmett-Teller (BET) surface area, pore volume and average pore sizes of the samples are summarized in Table 1. As calculated by the BET method, carbon aerogel samples CA_3_ and CA_4_ gave rise to a BET surface area of around 240 m^2^·g^−1^ and possessed a relatively smaller average pore size compared with samples CA_1_ and CA_2_, which demonstrated that the presence of water during hydrothermal treatment was one of the key point for preparation of carbon aerogel with high specific surface area. In addition, it is well known that the mercury porosimetry determines larger pores that are not within the detection range of nitrogen adsorption [16]. Therefore, mercury intrusion porosimeter (MIP) was also used for characterizing pore diameter distribution and size in porous materials as shown in Table 1 and Figure 4. It was noted that the pore diameter calculated on the basis of MIP data for CAs showed a pronounced increase as compared with that from nitrogen adsorption, which demonstrated that large amounts of mesopores and macropores were also present in the prepared carbon aerogels. The intrinsic hydrophobicity of an adsorbent is critical to achieve water-oil separation. Therefore, the hydrophobicity of carbon aerogel samples was characterized using contact angle as shown in Figure 5. The results indicated that all the carbon aerogel samples exhibited hydrophobic properties, in which the sample CA_3_ showed strongest hydrophobicity with a contact angle of 133°. Therefore, it could be concluded that CAs had great potential for efficient removal of oil spillage and chemical leakage due to their high porosity and excellent hydrophobicity.

### 2.5. Absorption Capacities for Oil and Organic Solvents

**Figure 6:**
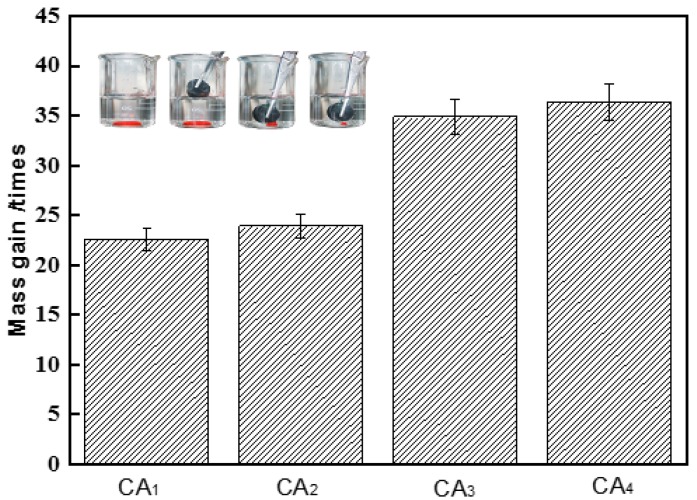
Adsorption capacity of carbon aerogels for gasoline. The error bar in each column indicates the standard deviation. Inset is the removal of chloroform dyed with Sudan red from underwater.

In order to quantitatively study the adsorption efficiency of CAs, the adsorption capacities of CA samples on gasoline were investigated. As shown in Figure 6 inset, a cylinder of CA sample was immersed into the water and put in contact with the chloroform droplet (stained with Sudan red), it could adsorb the chloroform completely and rapidly from the water. No water in the saturated CAs was found, which exhibited high selective adsorption for the organic solvents. The adsorption capacities of CA samples on gasoline indicated that samples CA_3_ and CA_4_ adsorbed gasoline at around 35 times its own weight, which exhibited superior adsorption capacities compared with zeolite and wool-based nonwoven adsorption materials [17,18].

**Figure 7:**
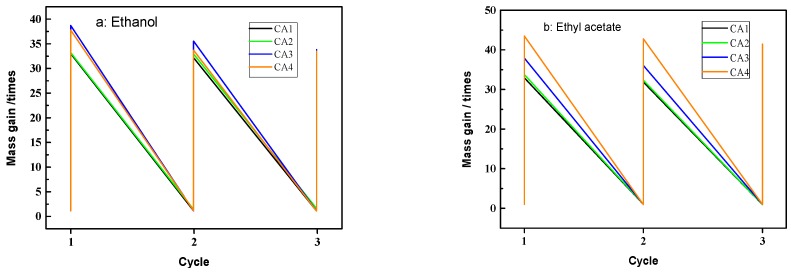
Recyclability of carbon aerogels for adsorption of ethanol (**a**) and ethyl acetate (**b**) with a distillation method.

The recyclability of adsorbents also plays important roles in pollution control and environmental protection. In the present study, distillation is employed to recover pristine CAs and harvest the pollutants. In general, after the liquids have been adsorbed by CAs, the saturated CAs was heated to 100 °C to release adhering liquid and the recovered CAs and various liquids were collected for recycling. The adsorption and distillation processes were repeated three times to investigate the reusability of CAs. As shown in Figure 7, there was no obvious decrease in adsorption capacities even after three cycles, which demonstrated that the carbon aerogels samples exhibited the stable performance. In addition, it was noted that the samples of CA_3_ and CA_4_ possessed higher adsorption capacities with 35–45 times its own weight for both organic solvents, which exhibited equal adsorption capacities compared with carbon aerogel obtained from winter melon [9]. Therefore, benefiting from the 3D porous structure, the low cost of the precursors, and the green route, the obtained flexible carbonaceous aerogels offered very attractive prospects and could be extended to the applications as adsorbents for environmental and ocean protection.

## 3. Materials and Methods

### 3.1. Materials

Fresh eggplant was purchased from local supermarket. All chemicals, ethanol, ethyl acetate, chloroform and Sudan red were purchased from Sigma-Aldrich Co., Ltd. (Shanghai, China) Gasoline was purchased from China Petroleum & Chemical Corporation (Beijing, China). Deionized water was used throughout the whole experiment.

### 3.2. Methods

#### 3.2.1. Preparation of Carbon Aerogels

After removing the external layer called epicarp, the fresh egglant was cut into an cylinder shape with appropriate volume. After that, the spongy and white tissue was placed into a Teflon-lined stainless steel autoclave. The autoclave was heated at 180 °C for 2 h under self-generated pressure in a closed system. After hydrothermal treatment, the obtained eggplant hydrogels (EH) were immersed in ethanol/hot water (1:1, v/v) for 2 days to remove soluble impurities. Then, the EH were freeze-dried, and placed in a tube furnace for pyrolyzing under N_2_ atmosphere. In order to evaluate the role of water in hydrothermal treatments, the fresh egglant was hydrothermally treated without and with water addition, and the corresponding eggplant hydrogels (EH) was noted as EH_1_ and EH_2_, respectively. After that, the obtained eggplant hydrogels were pyrolyzed with different pyrolyzing procedures. For the pyrolyzing prodedures (1), the temperature was raised to 250 °C and held for 1 h, and then temperature was raised to 800 °C and held for 1 h. The pyrolyzing prodedures (2) were same with procedures (1) except that the temperature was firstly raised to 400 °C and held for 1 h. All the pyrolyzing processes were all carried out under N_2_ atmosphere. The pyrolyzed eggplant carbon aerogels (CAs) from EH_1_ were named as CA_1_ and CA_2_, while that from EH_2_ were noted as CA_3_ and CA_4_, respectively. Moreover, the fresh eggplant was freezedried, and it was named as Ec. The treatment methods and heating procedures are shown in Table 2.

#### 3.2.2. Characterizations

**Figure 8:**
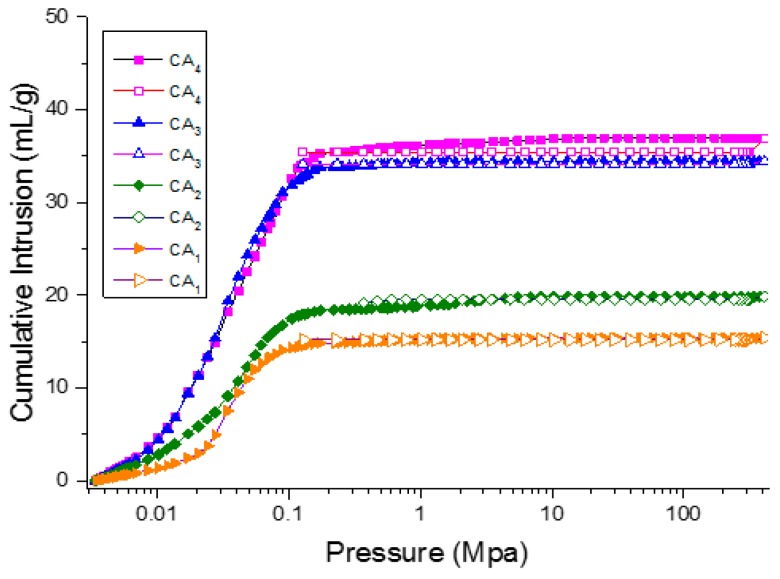
Mercury cumulative intrusion (filled dots) and extrusion curves (hollow dots) of carbon aerogels.

Scanning electron microscopy (SEM) micrographs were characterized with a Hitachi SU8010 scanning electron microscope (Hitachi High Tech Co., Tokyo, Japan) using an accelerating voltage of 5 kV. Fourier-transform infrared spectroscopy (FT-IR) analysis was measured by a Tensor 27 infrared spectrum instrument (German Bruker, Karlsruhe, Germany). The scan range was 400–4000 cm^−1^ and the resolution rate was 2 cm^−1^. The water contact angle of CAs was measured on a automatic video-based optical contact angle with drop contour analysis system (OCA15Pro, Beijing Eastern-Dataphy Instruments Co., Ltd., Beijing, China) and the measurement procedures was consistent with the previously published literature [19]. The Brunauer-Emmett-Teller (BET) and Barrett-Joyner-Halenda (BJH) measurements were performed by Micromeritics Tristar II 3020 analyzer (Micromeritics Instrument Corporation, Norcross, GA, USA). Mercury intrusion porosimetry (MIP) (AutoPore IV 9500 mercury porosimeter, Micromeritics Instrument Corporation, Norcross, GA, USA) was used to analyze the average pore diameter and size distribution of samples. For each experiment, about 0.3 g of sample was charged into a calibrated penetrometer and the amount of mercury intruded with increasing and then decreasing pressure was measured as shown in Figure 8. The physical properties included the contact angle between the mercury and the material, as well as surface tension of mercury were 130° and 485 dyn/cm, respectively. Moreover, the porosity, calculated from real density and apparent density, is presented in Table 1. Thermogravimetric analysis (TGA) test was carried out on a Q600SDT instrument (Thermal Analysis, New Castle, DE, USA). Approximately 10 mg of samples was used with heating rate of 10 °C/min at temperature range from 25 to 800 °C in nitrogen environment.

#### 3.2.3. Adsorption of Oils and Organic Solvents and Reusability

The adsorption capacities for oil and organic solvents was based on a calculation method proposed by Li [9]. Briefly, a cylinder sample of CA was placed in contact with the organic solvents or oils for 10 s, and then it was taken out for mass measurement. The mass of CA aerogel before and after adsorption was recorded for calculating the mass gain. The recycling of adsorbents is also a key parameter for evaluating the potential application in environmental protection. In order to evaluate the reusability of adsorbents, the adsorption and distillation processes were carried out at room temperature and 100 °C to regenerate the CA adsorbents, respectively.

## 4. Conclusions

In summary, the effect of presence of water during hydrothermal treatment and holding temperature during post-pyrolysis process had been investigated for the preparation of carbon aerogels using eggplant as raw material. The results showed that the presence of water or not during hydrothermal treatment was the key point for preparation of CA samples with outstanding physical properties. With the addition of water in hydrothermal process, the carbon aerogels with higher surface area and stronger hydrophobicity were obtained and they exhibited superior adsorption capacities for both oil and organic solvents compared with that fabricated without the presence of water during hydrothermal treatment. However, the holding temperature during post-pyrolysis exhibited insignificant influence on the physical properties of CAs. Under the optimum conditions, the prepared CAs exhibited outstanding adsorption capacities with 35–45 times its own weight for organic solvents and oil. Moreover, it possessed noteworthy reusability as the intrinsic adsorption capacities were almost maintained after three cycles. With a combination of low-cost biomass as raw materials, green preparation process, and excellent hydrophobicity, CAs was highly promising as an economic, efficient, and safe adsorbent for environmental and ocean protection.

## Figures and Tables

**Table 1:** Physical characteristics of carbon aerogels.

Samples	Nitrogen Adsorption			Mercury Intrusion Porosimetry	
	Surface Area (m^2^·g^−1^)	Pore Volume (cm^3^·g^−1^)	Pore Diameter (nm)	Average Pore Diameter (nm)	Total Porosity (%)
CA_1_	43	0.07	6.72	5968	88
CA_2_	48	0.08	8.72	6185	89
CA_3_	239	0.14	3.65	10,751	92
CA_4_	249	0.16	2.55	16,192	94

**Table 2:** Treatment methods and pyrolyzing procedures for preparing carbon aerogels.

Hydrothermal Treatment	Carbonization	
	Pyrolyzing Procedures (1) ^c^	Pyrolyzing Procedures (2) ^d^
EH_1_ **^a^**	CA_1_	CA_2_
EH_2_ **^b^**	CA_3_	CA_4_

**^a^** without water addition during hydrothermal treatment; **^b^** with water addition during hydrothermal treatment; **^c^** the temperature was raised to 250 °C and held for 1 h, and then the temperature was raised to 800 °C and held for 1 h under N_2_ atmosphere; **^d^** the temperature was raised to 400 °C and held for 1 h, and then the temperature was raised to 800 °C and held for 1 h under N_2_ atmosphere.

## References

[1] Bi H.C., Xie X., Yin K.B., Zhou Y.L., Wan S., He L.B., Xu F., Banhart F., Sun L.T., Ruoff R.S. (2012). Spongy graphene as a highly efficient and recyclable sorbent for oils and organic solvents. Adv. Funct. Mater..

[2] Bi H.C., Yin Z.Y., Cao X.H., Xie X., Tan C.L., Huang X., Chen B., Chen F.T., Yang Q.L., Bu X.Y. (2013). Carbon fiber aerogel made from raw cotton: A novel, efficient and recyclable sorbent for oils and organic solvents. Adv. Mater..

[3] Gui X.C., Zeng Z.P., Lin Z.Q., Gan Q.M., Xiang R., Zhu Y., Cao A.Y., Tang Z.K. (2013). Magnetic and highly recyclable macroporous carbon nanotubes for spilled oil sorption and separation. ACS Appl. Mater. Inter..

[4] Zhang X.Y., Li Z., Liu K.S., Jiang L. (2013). Bioinspired multifunctional foam with self-cleaning and oil/water separation. Adv. Funct. Mater..

[5] Chen N., Pan Q.M. (2013). Versatile fabrication of ultralight magnetic foams and application for oil-water separation. ACS Nano.

[6] Nguyen D.D., Tai N.H., Lee S.B., Kuo W.S. (2012). Superhydrophobic and superoleophilic properties of graphene-based sponges fabricated using a facile dip coating method. Energy Environ. Sci..

[7] Wu Z.Y., Li C., Liang H.W., Chen J.F., Yu S.H. (2013). Ultralight, flexible, and fire-resistant carbon nanofiber aerogels from bacterial cellulose. Angew. Chem. Int. Ed..

[8] Shin M.K., Oh J., Lima M., Kozlov M.E., Kim S.J., Baughman R.H. (2010). Elastomeric conductive composites based on carbon nanotube forests. Adv. Mater..

[9] Li Y.Q., Samad Y.A., Polychronopoulou K., Alhassan S.M., Liao K. (2014). Carbon aerogel from winter melon for highly efficient and recyclable oils and organic solvents absorption. ACS Sustain. Chem. Eng..

[10] Wu X.L., Wen T., Guo H.L., Yang S.B., Wang X.K., Xu A.W. (2013). Biomass-derived sponge-like carbonaceous hydrogels and aerogels for supercapacitors. ACS Nano.

[11] Titirici M.M., Thomas A., Yu S.H., Muller J.O., Antonietti M. (2007). A direct synthesis of mesoporous carbons with bicontinuous pore morphology from crude plant material by hydrothermal carbonization. Chem. Mater..

[12] Zia-ur-Rehman Z., Islam M., Shah W.H. (2003). Effect of microwave and conventional cooking on insoluble dietary fibre components of vegetables. Food Chem..

[13] Yuan T.Q., Xu F., He J., Sun R.C. (2010). Structural and physico-chemical characterization of hemicelluloses from ultrasound-assisted extractions of partially delignified fast-growing poplar wood through organic solvent and alkaline solutions. Biotechnol. Adv..

[14] Zhang X.M., Meng L.Y., Xu F., Sun R.C. (2011). Pretreatment of partially delignified hybrid poplar for biofuels production: Characterization of organosolv hemicelluloses. Ind. Crop. Prod..

[15] El Ouaqoudi F.Z., El Fels L., Winterton P., Lemee L., Ambles A., Hafidi M. (2014). Study of humic acids during composting of ligno-cellulose waste by Infra-red spectroscopic and thermogravimetric/thermal differential analysis. Compos. Sci. Util..

[16] Volzone C., Zagorodny N. (2014). Mercury intrusion porosimetry (MIP) study of archaeological pottery from Hualfin Valley, Catamarca, Argentina. Appl. Clay Sci..

[17] Sakthivel T., Reid D.L., Goldstein I., Hench L., Seal S. (2013). Hydrophobic high surface area zeolites derived from fly ash for oil spill remediation. Environ. Sci. Technol..

[18] Radetic M., Ilic V., Radojevic D., Miladinovic R., Jocic D., Jovancic P. (2008). Efficiency of recycled wool-based nonwoven material for the removal of oils from water. Chemosphere.

[19] Drelich J. (2013). Guidelines to measurements of reproducible contact angles using a sessile-drop technique. Surf. Innov..

